# Dimethyl [1-(1-allyl-5-iodo-1*H*-indol-3-yl)-3-hydroxy­prop­yl]phospho­nate

**DOI:** 10.1107/S1600536807068171

**Published:** 2008-01-09

**Authors:** Ying-Cen Guo, Xu-Fan Wang, Yu Ding

**Affiliations:** a Key Laboratory of Pesticides and Chemical Biology of Ministry of Education, College of Chemistry, Central China Normal University, Wuhan 430079, People’s Republic of China

## Abstract

In the title compound, C_16_H_21_INO_4_P, the mol­ecular structure is stabilized by a weak intra­molecular C—H⋯O hydrogen-bond inter­action. The crystal packing is stabilized by strong inter­molecular O—H ⋯ O hydogen-bonding inter­actions to form a zigzag packing arrangement.

## Related literature

For asymmetric synthesis of phospho­rus compounds, see: Carlone *et al.* (2007 ▶); Yang, *et al.* (2007 ▶); Ibrahem *et al.* (2007 ▶). For related structures, see: Sonar *et al.* (2006 ▶); Chen *et al.* (2007 ▶); Butcher *et al.* (2007 ▶). For related literature, see: Allen *et al.* (1989 ▶); Horiguchi & Kandatsu (1959 ▶).
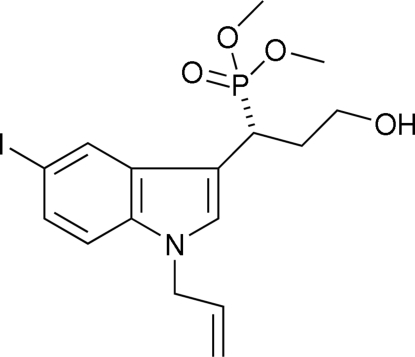


## Experimental

### 

#### Crystal data

C_16_H_21_INO_4_P
*M* *_r_* = 449.21
Orthorhombic, 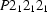

*a* = 7.9983 (4) Å
*b* = 10.1295 (6) Å
*c* = 22.3722 (12) Å
*V* = 1812.57 (17) Å^3^
*Z* = 4
Mo *K*α radiation
μ = 1.87 mm^−1^
*T* = 294 (2) K
0.20 × 0.10 × 0.10 mm

#### Data collection

Bruker SMART CCD area-detector diffractometer
Absorption correction: multi-scan (*SADABS*; Sheldrick, 1997 ▶) *T*_min_ = 0.706, *T*_max_ = 0.835
10836 measured reflections
3564 independent reflections
3314 reflections with *I* > 2σ(*I*)
*R*_int_ = 0.051

#### Refinement

*R*[*F* ^2^ > 2σ(*F* ^2^)] = 0.042
*wR*(*F* ^2^) = 0.102
*S* = 1.04
3564 reflections
211 parameters
H-atom parameters constrained
Δρ_max_ = 0.69 e Å^−3^
Δρ_min_ = −0.30 e Å^−3^
Absolute structure: Flack (1983 ▶), 1503 Freidel pairs
Flack parameter: 0.00 (1)

### 

Data collection: *SMART* (Bruker, 2001 ▶); cell refinement: *SAINT* (Bruker, 2001 ▶); data reduction: *SAINT*; program(s) used to solve structure: *SHELXS97* (Sheldrick, 2008 ▶); program(s) used to refine structure: *SHELXL97* (Sheldrick, 2008 ▶); molecular graphics: *SHELXTL* (Bruker, 2001 ▶); software used to prepare material for publication: *SHELXTL*.

## Supplementary Material

Crystal structure: contains datablocks I, global. DOI: 10.1107/S1600536807068171/at2520sup1.cif

Structure factors: contains datablocks I. DOI: 10.1107/S1600536807068171/at2520Isup2.hkl

Additional supplementary materials:  crystallographic information; 3D view; checkCIF report

## Figures and Tables

**Table 1:** Hydrogen-bond geometry (Å, °)

*D*—H⋯*A*	*D*—H	H⋯*A*	*D*⋯*A*	*D*—H⋯*A*
C12—H12⋯O1	0.98	2.47	2.873 (7)	104
O1—H1⋯O4^i^	0.82	1.92	2.733 (6)	174

Symmetry code: (i) 
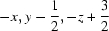
.

**Fig. 1.**
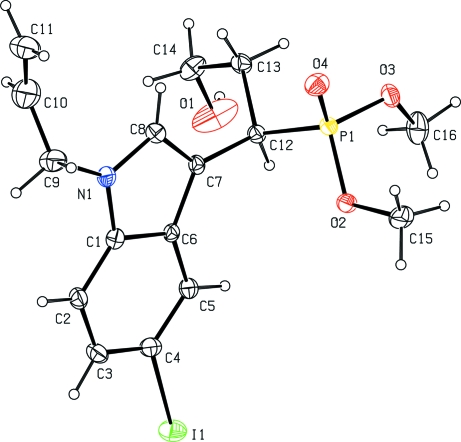
The molecular structure of (I), showing the atom-labelling scheme and 50% probability displacement ellipsoids.

**Fig. 2.**
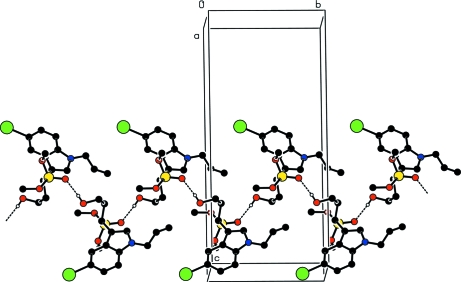
The packing view of the title compound (I) along a axis, showing intermolecular hydrogen bonds as dashed lines. H atoms not involved in hydrogen bonding have been omitted for clarity..

**Fig. 3.**
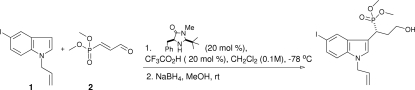
The reaction scheme.

## supplementary crystallographic information

### Comment

Phosphorus has been recognized as an essential structural constituent of many biomolecules and a crucial element in many biological transformations (Horiguchi & Kandatsu,1959; Allen *et al.* 1989). With the advances in the development of chiral catalysts, asymmetric synthesis of some phosphorus compounds has been well documented in literatures (Carlone *et al.*, 2007; Yang, *et al.*, 2007; Ibrahem *et al.*, 2007). As part of our ongoing project on enantioselective organocatalysis, a series of α-indolyl phosphonates have been highly enantioselectively synthesized *via* Friedel-Crafts alkylation of substituted indoles with (*E*)-dialkyl 3-oxoprop-1-enylphosphonate using MacMillan's imidazolidinone catalysts. The title compound (I) was synthesized and its crystal structure is presented here.

As seen in Fig. 1, the pyrrole plane (C1/C6—C8/N1) and the phenyl ring (C1—C6) are coplanar with each other with a dihedral angle between their mean planes of 2.2 (24)° in the molecule of the title compound (I), and the molecular structure is stablized by a week C12—H12 ··· O1 intramolecular hydrogen bonding interaction (Table 1).

The crystal packing (Fig. 2) is stabilized by strong intermolecular O—H ··· O hydogen bonds interaction (Table 1) to form a zigzag packing arrangement. Dipole–dipole and van der Waals interactions are also effective in the molecular packing in the crystal structure.

### Experimental

The MacMillan's imidazolidinone catalysts (Fig 3) (0.02 mmol) and TFA (0.02 mmol) were stirred in 1 ml dichloromethane for 5 min at 195 K, then (*E*)-dialkyl 3-oxoprop-1-enylphosphonate 2 (0.1 mmol) was added and stirred for 15 min. And then 1-allyl-5-iodo-1*H*-indole 1 (0.11 mmol) was added. After the mixture was stirred for 48 h, the solvent (dichloromethane) was removed under reduced pressure at room temperature, and then the residue was added to a stirring solution of sodium borohydride (18.6 mg, 0.5 mmol, 5 equiv) in methanol (3.0 ml). After 15 min, the reaction was quenched with saturated aqueous NaHCO_3_ and extracted with CH_2_Cl_2_. The combined organic layer was washed with saturated solutions of NaHCO_3_ and NaCl and then dried over with Na_2_SO_4_. The combined organic layer was concentrated *in vacuo* and the product was purified by flash column chromatography on silica gel (ethyl acetate, *R*_f_ = 0.4), (14.3 mg, 48% yield). Compound 5 m: yellow solid; tr (minor) = 53.8 min, tr (major) = 59.5 min (Chiralcel AD—H, λ = 254 nm, 5% i-PrOH / hexanes, flow rate = 1.0 ml/min). ^1^H NMR (400 MHz, CDCl_3_) δ 2.10–2.18 (m, 1H), 2.28–2.35 (m, 1H), 2.59 (br, 1H), 3.49 (d, J = 10.8 Hz, 3H), 3.73 (d, J = 10.8 Hz, 3H), 3.50–3.75 (m, 3H), 4.67 (d, J = 5.2 Hz, 2H), 5.00 (d, J = 16.8 Hz, 1H), 5.19 (d, J = 10.4 Hz, 1H), 5.90–6.02 (m, 1H), 7.05–7.47 (m, 3H), 7.98 (s, 1H). ^13^C NMR (100 MHz, CDCl_3_) δ 29.9, 31.3, 33.3, 48.9, 52.67, 52.75, 53.4, 53.5, 59.7, 59.8, 83.0, 107.9, 111.8, 117.5, 127.9, 128.5, 129.5, 130.2, 132.8, 135.2.; MS (EI) m/*z* 449 (*M*^+^), 450 (*M*^+^+1), 451 (*M*^+^+2). Elemental Analysis calculated for C_16_H_21_INO_4_P: C 42.78, H 4.71, N 3.12%. Found: C 42.58, H 4.94, N 3.99%. [α]23D = -22.62 (C=2.35, CHCl_3_) (after recrystallization). Single crystals of (I) suitable for X-ray diffraction were obtained by slow evaporation of a CHCl_3_ solution.

### Refinement

All H atoms were positioned geometrically and treated as riding on their parent atoms, with *C*—*H*(methyl) = 0.96 Å, *C*—*H*(methylene) = 0.97 Å, C—H(methine) = 0.98 Å, C—H(aromatic) = 0.93 Å and O—H = 0.82 Å, and with *U*_iso_(H) =1.5*U*_eq_(C_methyl_,*O*) and 1.2*U*_eq_(C_aromatic_,*C*_methylene_,*C*_methineylene_).

### Crystal data

**Table:**

C_16_H_21_INO_4_P	*F*_000_ = 896
*M**_r_* = 449.21	*D*_x_ = 1.646 Mg m^−^^3^
Orthorhombic, *P*2_1_2_1_2_1_	Mo *K*α radiation λ = 0.71073 Å
Hall symbol: P 2ac 2ab	Cell parameters from 5073 reflections
*a* = 7.9983 (4) Å	θ = 2.2–25.6º
*b* = 10.1295 (6) Å	µ = 1.87 mm^−^^1^
*c* = 22.3722 (12) Å	*T* = 294 (2) K
*V* = 1812.57 (17) Å^3^	Block, colourless
*Z* = 4	0.20 × 0.10 × 0.10 mm

### Data collection

**Table:**

Bruker SMART CCD area-detector diffractometer	3314 reflections with *I* > 2σ(*I*)
Monochromator: graphite	*R*_int_ = 0.051
*T* = 294(2) K	θ_max_ = 26.0º
phi and ω scans	θ_min_ = 1.8º
Absorption correction: multi-scan(SADABS; Sheldrick, 1997)	*h* = −7→9
*T*_min_ = 0.706, *T*_max_ = 0.835	*k* = −11→12
10836 measured reflections	*l* = −27→27
3564 independent reflections	

### Refinement

**Table:**

Refinement on *F*^2^	Hydrogen site location: inferred from neighbouring sites
Least-squares matrix: full	H-atom parameters constrained
*R*[*F*^2^ > 2σ(*F*^2^)] = 0.042	*w* = 1/[σ^2^(*F*_o_^2^) + (0.0573*P*)^2^ + 0.6855*P*] where *P* = (*F*_o_^2^ + 2*F*_c_^2^)/3
*wR*(*F*^2^) = 0.102	(Δ/σ)_max_ < 0.001
*S* = 1.04	Δρ_max_ = 0.69 e Å^−^^3^
3564 reflections	Δρ_min_ = −0.30 e Å^−^^3^
211 parameters	Extinction correction: none
Primary atom site location: structure-invariant direct methods	Absolute structure: Flack (1983), 1503 Freidel pairs
Secondary atom site location: difference Fourier map	Flack parameter: 0.00 (1)

### Special details

**Table:**

Geometry. All e.s.d.'s (except the e.s.d. in the dihedral angle between two l.s. planes) are estimated using the full covariance matrix. The cell e.s.d.'s are taken into account individually in the estimation of e.s.d.'s in distances, angles and torsion angles; correlations between e.s.d.'s in cell parameters are only used when they are defined by crystal symmetry. An approximate (isotropic) treatment of cell e.s.d.'s is used for estimating e.s.d.'s involving l.s. planes.
Refinement. Refinement of *F*^2^ against ALL reflections. The weighted *R*-factor *wR* and goodness of fit *S* are based on *F*^2^, conventional *R*-factors *R* are based on *F*, with *F* set to zero for negative *F*^2^. The threshold expression of *F*^2^ > σ(*F*^2^) is used only for calculating *R*-factors(gt) *etc*. and is not relevant to the choice of reflections for refinement. *R*-factors based on *F*^2^ are statistically about twice as large as those based on *F*, and *R*- factors based on ALL data will be even larger.

### Fractional atomic coordinates and isotropic or equivalent isotropic displacement parameters (Å^2^)

**Table:**

	*x*	*y*	*z*	*U*_iso_*/*U*_eq_	
I1	−0.17244 (5)	−0.23507 (4)	1.053601 (16)	0.06002 (15)	
C1	−0.1488 (6)	0.1982 (4)	0.96323 (18)	0.0340 (9)	
C2	−0.2151 (6)	0.1860 (5)	1.0201 (2)	0.0408 (11)	
H2	−0.2575	0.2587	1.0404	0.049*	
C3	−0.2158 (7)	0.0611 (6)	1.0455 (2)	0.0460 (12)	
H3	−0.2580	0.0495	1.0838	0.055*	
C4	−0.1542 (7)	−0.0467 (5)	1.0142 (2)	0.0414 (11)	
C5	−0.0842 (6)	−0.0348 (5)	0.95801 (19)	0.0364 (10)	
H5	−0.0414	−0.1081	0.9382	0.044*	
C6	−0.0794 (5)	0.0896 (4)	0.93175 (17)	0.0299 (9)	
C7	−0.0233 (5)	0.1394 (4)	0.87529 (18)	0.0303 (9)	
C8	−0.0615 (5)	0.2707 (5)	0.87450 (19)	0.0365 (10)	
H8	−0.0395	0.3272	0.8427	0.044*	
C9	−0.1972 (10)	0.4394 (5)	0.9422 (2)	0.0627 (17)	
H9A	−0.2818	0.4309	0.9731	0.075*	
H9B	−0.1050	0.4893	0.9591	0.075*	
C10	−0.2659 (14)	0.5131 (9)	0.8942 (4)	0.105 (3)	
H10	−0.3574	0.4747	0.8753	0.126*	
C11	−0.2201 (13)	0.6240 (7)	0.8734 (3)	0.089 (3)	
H11A	−0.1294	0.6679	0.8902	0.107*	
H11B	−0.2772	0.6613	0.8414	0.107*	
C12	0.0602 (6)	0.0611 (5)	0.82594 (19)	0.0324 (9)	
H12	0.0481	−0.0329	0.8355	0.039*	
C13	−0.0169 (6)	0.0843 (5)	0.76336 (19)	0.0414 (11)	
H13A	−0.0210	0.1785	0.7555	0.050*	
H13B	0.0547	0.0443	0.7334	0.050*	
C14	−0.1891 (8)	0.0284 (6)	0.7576 (2)	0.0541 (14)	
H14A	−0.2652	0.0777	0.7829	0.065*	
H14B	−0.2268	0.0374	0.7166	0.065*	
C15	0.5194 (7)	0.0595 (7)	0.9005 (3)	0.0577 (15)	
H15A	0.5880	0.0042	0.8757	0.087*	
H15B	0.5363	0.0367	0.9417	0.087*	
H15C	0.5494	0.1503	0.8943	0.087*	
C16	0.3565 (9)	−0.1289 (6)	0.7724 (3)	0.0686 (18)	
H16A	0.4059	−0.1626	0.8084	0.103*	
H16B	0.4166	−0.1620	0.7384	0.103*	
H16C	0.2419	−0.1566	0.7701	0.103*	
N1	−0.1369 (5)	0.3078 (4)	0.92702 (16)	0.0379 (9)	
O1	−0.1922 (9)	−0.1050 (5)	0.7740 (3)	0.112 (2)	
H1	−0.2269	−0.1492	0.7459	0.168*	
O2	0.3445 (4)	0.0404 (3)	0.88494 (12)	0.0386 (7)	
O3	0.3641 (4)	0.0129 (4)	0.77276 (15)	0.0494 (9)	
O4	0.3217 (5)	0.2376 (3)	0.81376 (16)	0.0521 (8)	
P1	0.28050 (14)	0.09837 (13)	0.82350 (5)	0.0338 (3)	

### Atomic displacement parameters (Å^2^)

**Table:**

	*U*^11^	*U*^22^	*U*^33^	*U*^12^	*U*^13^	*U*^23^
I1	0.0725 (3)	0.0485 (2)	0.0591 (2)	−0.00777 (18)	0.00460 (18)	0.01635 (16)
C1	0.032 (2)	0.034 (2)	0.036 (2)	0.0011 (18)	−0.0051 (17)	−0.0074 (16)
C2	0.037 (2)	0.050 (3)	0.036 (2)	0.006 (2)	0.0039 (19)	−0.006 (2)
C3	0.047 (3)	0.058 (3)	0.032 (2)	0.000 (2)	0.008 (2)	0.001 (2)
C4	0.041 (3)	0.045 (3)	0.038 (2)	−0.003 (2)	−0.003 (2)	0.0070 (19)
C5	0.032 (2)	0.037 (2)	0.040 (3)	−0.0025 (19)	0.0006 (19)	−0.0012 (19)
C6	0.024 (2)	0.038 (2)	0.028 (2)	0.0039 (18)	−0.0029 (16)	−0.0024 (17)
C7	0.023 (2)	0.038 (2)	0.030 (2)	0.0002 (18)	−0.0002 (16)	−0.0048 (17)
C8	0.037 (2)	0.038 (2)	0.035 (2)	−0.003 (2)	0.0003 (18)	−0.0033 (19)
C9	0.104 (5)	0.039 (3)	0.045 (3)	0.022 (3)	0.005 (4)	−0.002 (2)
C10	0.141 (8)	0.083 (6)	0.090 (6)	0.057 (6)	0.025 (5)	0.008 (5)
C11	0.157 (8)	0.053 (4)	0.058 (4)	0.043 (5)	0.035 (4)	0.010 (3)
C12	0.034 (2)	0.032 (2)	0.031 (2)	0.0014 (19)	0.0026 (18)	−0.0003 (18)
C13	0.042 (3)	0.054 (3)	0.028 (2)	0.002 (2)	−0.0016 (19)	−0.007 (2)
C14	0.056 (3)	0.051 (3)	0.055 (3)	0.000 (3)	−0.026 (3)	−0.006 (2)
C15	0.038 (3)	0.080 (4)	0.056 (3)	−0.006 (3)	−0.013 (2)	0.014 (3)
C16	0.060 (4)	0.062 (4)	0.083 (4)	0.011 (3)	0.004 (3)	−0.029 (3)
N1	0.042 (2)	0.035 (2)	0.0367 (19)	0.0083 (17)	0.0030 (16)	−0.0041 (15)
O1	0.153 (6)	0.054 (3)	0.129 (5)	−0.034 (4)	−0.088 (4)	0.005 (3)
O2	0.0300 (16)	0.0523 (19)	0.0334 (15)	−0.0042 (16)	−0.0025 (14)	0.0044 (13)
O3	0.035 (2)	0.071 (3)	0.0416 (18)	0.0069 (17)	0.0066 (15)	−0.0039 (17)
O4	0.0469 (19)	0.047 (2)	0.062 (2)	−0.007 (2)	−0.0022 (16)	0.0139 (16)
P1	0.0292 (6)	0.0419 (7)	0.0304 (5)	0.0007 (5)	0.0017 (4)	0.0023 (5)

### Geometric parameters (Å, °)

**Table:**

I1—C4	2.107 (5)	C11—H11B	0.9300
C1—N1	1.378 (6)	C12—C13	1.548 (6)
C1—C2	1.383 (6)	C12—P1	1.803 (4)
C1—C6	1.419 (6)	C12—H12	0.9800
C2—C3	1.387 (7)	C13—C14	1.495 (8)
C2—H2	0.9300	C13—H13A	0.9700
C3—C4	1.387 (7)	C13—H13B	0.9700
C3—H3	0.9300	C14—O1	1.400 (7)
C4—C5	1.382 (6)	C14—H14A	0.9700
C5—C6	1.391 (7)	C14—H14B	0.9700
C5—H5	0.9300	C15—O2	1.455 (6)
C6—C7	1.432 (6)	C15—H15A	0.9600
C7—C8	1.365 (7)	C15—H15B	0.9600
C7—C12	1.514 (6)	C15—H15C	0.9600
C8—N1	1.374 (5)	C16—O3	1.438 (7)
C8—H8	0.9300	C16—H16A	0.9600
C9—C10	1.419 (10)	C16—H16B	0.9600
C9—N1	1.458 (6)	C16—H16C	0.9600
C9—H9A	0.9700	O1—H1	0.8200
C9—H9B	0.9700	O2—P1	1.580 (3)
C10—C11	1.270 (12)	O3—P1	1.576 (4)
C10—H10	0.9300	O4—P1	1.464 (4)
C11—H11A	0.9300		
			
N1—C1—C2	129.7 (4)	C7—C12—H12	107.8
N1—C1—C6	107.8 (4)	C13—C12—H12	107.8
C2—C1—C6	122.5 (4)	P1—C12—H12	107.8
C1—C2—C3	117.3 (4)	C14—C13—C12	112.8 (4)
C1—C2—H2	121.3	C14—C13—H13A	109.0
C3—C2—H2	121.3	C12—C13—H13A	109.0
C4—C3—C2	120.7 (4)	C14—C13—H13B	109.0
C4—C3—H3	119.7	C12—C13—H13B	109.0
C2—C3—H3	119.7	H13A—C13—H13B	107.8
C5—C4—C3	122.2 (4)	O1—C14—C13	111.1 (5)
C5—C4—I1	119.2 (4)	O1—C14—H14A	109.4
C3—C4—I1	118.5 (3)	C13—C14—H14A	109.4
C4—C5—C6	118.4 (4)	O1—C14—H14B	109.4
C4—C5—H5	120.8	C13—C14—H14B	109.4
C6—C5—H5	120.8	H14A—C14—H14B	108.0
C5—C6—C1	118.8 (4)	O2—C15—H15A	109.5
C5—C6—C7	134.4 (4)	O2—C15—H15B	109.5
C1—C6—C7	106.7 (4)	H15A—C15—H15B	109.5
C8—C7—C6	106.5 (4)	O2—C15—H15C	109.5
C8—C7—C12	126.8 (4)	H15A—C15—H15C	109.5
C6—C7—C12	126.7 (4)	H15B—C15—H15C	109.5
C7—C8—N1	110.8 (4)	O3—C16—H16A	109.5
C7—C8—H8	124.6	O3—C16—H16B	109.5
N1—C8—H8	124.6	H16A—C16—H16B	109.5
C10—C9—N1	115.6 (5)	O3—C16—H16C	109.5
C10—C9—H9A	108.4	H16A—C16—H16C	109.5
N1—C9—H9A	108.4	H16B—C16—H16C	109.5
C10—C9—H9B	108.4	C8—N1—C1	108.2 (4)
N1—C9—H9B	108.4	C8—N1—C9	126.6 (4)
H9A—C9—H9B	107.4	C1—N1—C9	125.2 (4)
C11—C10—C9	129.1 (11)	C14—O1—H1	109.5
C11—C10—H10	115.5	C15—O2—P1	118.1 (3)
C9—C10—H10	115.5	C16—O3—P1	122.4 (4)
C10—C11—H11A	120.0	O4—P1—O3	109.0 (2)
C10—C11—H11B	120.0	O4—P1—O2	114.5 (2)
H11A—C11—H11B	120.0	O3—P1—O2	106.55 (19)
C7—C12—C13	113.8 (4)	O4—P1—C12	115.2 (2)
C7—C12—P1	110.1 (3)	O3—P1—C12	108.7 (2)
C13—C12—P1	109.3 (3)	O2—P1—C12	102.26 (19)
			
N1—C1—C2—C3	177.8 (5)	C7—C12—C13—C14	−69.1 (6)
C6—C1—C2—C3	−1.6 (7)	P1—C12—C13—C14	167.4 (4)
C1—C2—C3—C4	−0.9 (7)	C12—C13—C14—O1	−53.2 (7)
C2—C3—C4—C5	2.5 (8)	C7—C8—N1—C1	−0.1 (5)
C2—C3—C4—I1	−176.4 (4)	C7—C8—N1—C9	−179.2 (5)
C3—C4—C5—C6	−1.5 (7)	C2—C1—N1—C8	−179.9 (5)
I1—C4—C5—C6	177.4 (3)	C6—C1—N1—C8	−0.5 (5)
C4—C5—C6—C1	−1.0 (6)	C2—C1—N1—C9	−0.8 (8)
C4—C5—C6—C7	−177.9 (5)	C6—C1—N1—C9	178.7 (5)
N1—C1—C6—C5	−176.9 (4)	C10—C9—N1—C8	35.3 (10)
C2—C1—C6—C5	2.6 (6)	C10—C9—N1—C1	−143.6 (7)
N1—C1—C6—C7	0.8 (5)	C16—O3—P1—O4	175.5 (4)
C2—C1—C6—C7	−179.7 (4)	C16—O3—P1—O2	51.4 (5)
C5—C6—C7—C8	176.3 (5)	C16—O3—P1—C12	−58.1 (5)
C1—C6—C7—C8	−0.8 (5)	C15—O2—P1—O4	−51.1 (5)
C5—C6—C7—C12	−3.0 (8)	C15—O2—P1—O3	69.5 (4)
C1—C6—C7—C12	179.9 (4)	C15—O2—P1—C12	−176.4 (4)
C6—C7—C8—N1	0.6 (5)	C7—C12—P1—O4	−58.6 (4)
C12—C7—C8—N1	179.9 (4)	C13—C12—P1—O4	67.1 (4)
N1—C9—C10—C11	−120.5 (9)	C7—C12—P1—O3	178.7 (3)
C8—C7—C12—C13	−47.8 (6)	C13—C12—P1—O3	−55.6 (4)
C6—C7—C12—C13	131.4 (5)	C7—C12—P1—O2	66.3 (3)
C8—C7—C12—P1	75.3 (5)	C13—C12—P1—O2	−168.0 (3)
C6—C7—C12—P1	−105.6 (4)		

### Hydrogen-bond geometry (Å, °)

**Table:**

*D*—H···*A*	*D*—H	H···*A*	*D*···*A*	*D*—H···*A*
C12—H12···O1	0.98	2.47	2.873 (7)	104
O1—H1···O4^i^	0.82	1.92	2.733 (6)	174

Symmetry codes: (i) −*x*, *y*−1/2, −*z*+3/2.
